# Providing substance use disorder treatment in correctional settings: knowledge gaps and proposed research priorities—overview and commentary

**DOI:** 10.1186/s13722-022-00351-0

**Published:** 2022-12-08

**Authors:** Nickolas D. Zaller, Margaret M. Gorvine, Jon Ross, Shannon Gwin Mitchell, Faye S. Taxman, David Farabee

**Affiliations:** 1 University of Arkansas for Medical Sciences, College of Public Health, Little Rock, AR USA; 2 TASC, Inc. (Treatment Alternatives for Safe Communities), Chicago, IL USA; 3 Friends Research Institute, Baltimore, MD USA; 4 George Mason University, Schar School of Policy and Government, Fairfax, VA USA; 5 New York University, School of Medicine, New York, NY USA

**Keywords:** Substance use disorder, Treatment, Criminal justice

## Abstract

This manuscript is the product of the authors’ discussions, literature overview, and consultation with experts in the field, and identifies important gaps in the evidence base for substance use disorder (SUD) treatment effectiveness within criminal justice (CJ) settings. Lacking from the extant literature are longitudinal investigations of treatment related outcomes during and after incarceration. Such studies could provide rich contextual data about treatment delivery and effectiveness across the CJ continuum, and would provide important insight into individual characteristics (e.g., motivation, treatment modality preferences, treatment completion rates, etc.) as well as institutional and environmental factors (e.g., appropriate staffing, space limitations for individual treatment sessions, distribution of medications, etc.). We also identified the importance of reproducibility within CJ research, and the unfortunate reality of too many single studies conducted in single (or relatively few) correctional facilities. Some of this has been because the studies designed to produce that evidence are not prioritized for funding, which has continually placed researchers in a position where we cannot make firm conclusions or recommendations based on available evidence. The importance of replicating the foundational studies in this field cannot be overstated. We hope this article spurs other researchers to join in the healthy process of questioning the existing state of the CJ-based SUD treatment research, what should be re-examined, and how we can lay a stronger foundation for the future.

## Introduction

Individuals involved in the criminal justice (CJ) system are disproportionately impacted by substance use, with estimates of substance use dependence or abuse among correctional populations ranging from 58 to 63% for individuals incarcerated in prisons and jails, respectively [5] as well as those on probation and/or parole [34]. Improving access to evidence-based substance use disorder (SUD) treatment in correctional settings is an urgent need. This manuscript was developed following several months of discussion among the authors and many other researchers in this field in response to a request for concrete clinical recommendations that would inform the development of criminal justice treatment guidelines by the American Society of Addiction Medicine (ASAM) which is the subject of another paper. As we endeavored to provide answers we all agreed upon, our scrutiny of the literature generated a much longer list of questions than answers. All of the authors provided their expert opinion based on decades of collective experience as researchers at the nexus of SUD and CJ involvement; and four of the authors (NZ, MG, JR, DF) conducted targeted searches based on all authors’ consensus to identify relevant literature to confirm the research gaps. We acknowledge that while we did not conduct a systematic review around SUD treatment within CJ settings, we believe that addressing the yet-answered questions we describe below would go a long way toward creating a more solid foundation for delivering effective SUD care to justice-involved patients.

Despite the known substantial need for SUD treatment among individuals who are involved in the justice system, there is a dearth of scientific evidence regarding effective treatments for this population. While researchers have outlined the key components of corrections-based treatment (broadly defined as including assessment, cognitive behavior treatment services and contingency management compliance strategies [9, 25], there are few empirical studies to support effective content for behavioral programming (e.g., type and extent of counseling and/or psychoeducation) (see Taxman [79] for a discussion). Drug treatments in non-CJ settings are delivered in a variety of formats (e.g., individual and group) and are composed of behavioral therapies such as cognitive-behavioral therapy or contingency management, and may include medication treatment for opioid use disorders as a stand-alone treatment or in combination with the behavioral therapies [54]. But we do not have sufficient evidence as to which of these formats are most appropriate in CJ-settings.

Previous research has demonstrated that the majority of SUD treatment offered in CJ settings involve psychoeducation or group counseling, both of which are more appropriate for people with low-threshold SUD [78]. Additionally, much of the recent scientific literature has been specific to opioid use disorder (OUD), for which effective medications exist to reduce disease severity and improve recovery related outcomes [3], whereas marijuana, cocaine, methamphetamines, and other substances do not have an FDA-approved medication.

Important to the discussion of SUD treatment modalities within correctional environments is the setting in which treatment is delivered. For example, state prisons may be conducive to more intensive SUD treatment services because individuals tend to be incarcerated for longer periods of time (e.g., years) than those in jail. Individuals in county jails are often incarcerated for relatively short periods of time, often weeks to months but sometimes only hours or days (and typically less than 1-year maximum), which makes SUD treatment assessment, planning, and delivery challenging. Understanding which specific treatment modalities are most appropriate for specific correctional contexts is a critical gap in our collective knowledge about SUD treatment effectiveness among CJ populations. Furthermore, organizational climate can facilitate or inhibit adoption and implementation of evidence-based SUD treatment services in correctional settings [64]. This has been well documented in the case of medications to treat OUD within correctional settings [43]. Additionally, prior research has documented a high level of stigma among correctional staff toward incarcerated individuals with behavioral health disorders, in general, and SUD, specifically [42]. Perceived stigma of MHD and SUD is common in correctional settings and a reliably negative predictor of treatment uptake and adherence [10, 16].

In this paper, we outline key areas in which sizable knowledge gaps exist with respect to SUD treatment effectiveness in correctional settings, including: how much treatment (dosage) is sufficient to achieve positive gains (enough); to what extent co-occurring disorders should be addressed; what are the appropriate goals of treatment; whether or not mandating treatment to those who don’t want it is worthwhile; and how evidence-based practice can be better represented through executive/legislative policy or action. We recognize the inherent challenges in conducting health services research within CJ settings [36]. However, the dearth of SUD treatment effectiveness research in prisons and jails makes issuing treatment recommendations challenging. Therefore, we outline key research priorities which will further advance our knowledge of what types of SUD treatments work and for which populations and in which settings.

## Methods

This manuscript was an outgrowth of an ongoing effort by ASAM to revise current SUD treatment guidelines for CJ settings. The first author (NZ) is a co-Chair of the committee convened by ASAM to revise these guidelines. The other coauthors are affiliated with the NIDA funded Justice Community Opioid Innovation Network (JCOIN), a national network of researchers and CJ and SUD practitioners working on implementation of medications to treat opioid use disorder (MOUD) in CJ settings. The ASAM committee identified a dearth of literature regarding evidence-based SUD treatment outcomes in CJ settings as a primary challenge for revising current guidelines for SUD treatment in CJ settings. We worked independently of the ASAM committee to highlight important areas where further research is needed. For each area discussed below, we conducted targeted literature searches in each area. We completed a review of EBSCOhost (CINAHL, PsychInfor, PsycArticles, Psychology & Behavioral Sciences Collection, SocINDEX, etc.), and Pub Med in our targeted searches to identify examples in the extant literature associated with each area. We then met as a group to review the state of knowledge and to reach consensus regarding the evidence-base, or lack thereof, associated with each area.

### SUD treatment duration, intensity and composition

#### How to assess needs for SUD treatment and ancillary services?

The prominent tools used to assess service needs among those in correctional settings—Level of Service Inventory-Revised (LSI-R), Correctional Offender Management Profiling for Alternative Sanctions (COMPAS), and Ohio Risk Assessment System (ORAS) which are typically criminal legal system risk and need assessment tools—can be costly (both in terms of fiscal requirements and staff burden) and time consuming. Costly, burdensome assessments may actually pose a barrier to treatment. A study examining service-need determinations based on scores from the LSI-R and COMPAS compared to results of four yes/no questions regarding need for education, vocational assistance, substance use treatment, and housing showed that the single-item indicators identified 70–90% of those flagged as having high need for these services on the LSI-R and COMPAS instruments [19]. The latter study was based on a relatively small sample, but the findings are consistent with other trends in the literature. A study by Smith et al. [75] compared results of a single-item screen for drug use (“How many times in the past year have you used an illegal drug or used a prescription medication for nonmedical reasons?”) with the results of the 10-item Drug Abuse Screening Test (DAST-10). The single item was 100% sensitive and 74% specific for the detection of a drug use disorder (as determined by the CIDI Substance Abuse Module)—almost identical to that of the DAST-10. Citing the time constraints in primary care settings, Smith et al. [75] advised the expanded use of simpler, more straightforward approaches to screen for potential drug problems. As the implementation of screening for SUDs in CJ settings increases, finding approaches that are simple, expeditious, and cost-effective are important considerations.

#### What is the effective dose–response?

The literature has long relied on the positive correlation between treatment retention and reductions in substance use, dating to the cohort studies of Drug Abuse Treatment Outcomes (DATOS), which found that those individuals who were in treatment 90-days or more tended to have reduced drug use and re-arrest relative to those with shorter treatment durations [73, 74]. DATOS was conducted in community-based treatment services and does not necessarily translate into services within correctional settings. Nonetheless, DATOS was instrumental to understanding factors that affect retention in treatment for 90 days or more.

We are only aware of one randomized controlled trial assessing *prescribed* treatment duration. That study, conducted by Rawson et al. [65], was conducted with individuals (N = 85) in a community-based treatment programs, and showed a slight improved effect on drug use outcomes for those randomized to longer treatment; however, few participants in either group successfully completed treatment (defined as having attended 75% of CBT treatment sessions: 12 CBT sessions within a 4-week period or 48 CBT sessions within a 16-week period). The study found that individuals who completed treatment, regardless of study condition, had greater odds of stimulant-free urines [65]. The above study was not based on a CJ sample, however, the results point to a common problem in the SUD and CJ treatment literature: the misattribution of treatment outcomes to programs rather than individual-level factors (e.g., ability/willingness to complete a prescribed treatment regimen as compared to severity of need). Why does the same modality have no effect in prison but large effects in the community? It is difficult to know if this is because of the unique attributes of the prison or community environment or because of individual factors (e.g., motivation to participate in treatment is a more important factor in the community than in jail or prison). Alternatively, it is possible that treatment is ineffective in both settings but that those who opt to enter and remain in aftercare (commonly ~ 10% of those who agreed to attend; [6] are more committed to recovery and the presumed effects of treatment are really the spoils of selection bias. Similarly, we lack research on the length of time individuals should receive medications for opioid use disorder (MOUD) to affect their long-term substance free or reduced substance use outcomes. Just as with clinical therapy, there is a need to have more research on the dose needed to achieve positive outcomes for therapies, whether they are pharmacological or behavioral in nature.

#### If medications are part of the treatment plan, what level of counseling support is needed to achieve optimal outcomes?

The requirement (for methadone) or recommendation (for naltrexone and buprenorphine) of counseling seems to be an intuitive complement to MOUD alone. It is worth noting that there is significant variation in counseling services for SUD and the legal requirements do not specify the nature, type, or length of services. As with the prior section describing treatment dose–response relationships, it is unclear the extent to which the nature and duration of counseling would provide additional benefit, especially among incarcerated populations. It is therefore unsurprising that the evidence that counseling improves outcomes over and beyond MOUD is mixed, with the strongest support derived from post-hoc analyses. In an experimental comparison (of a non-CJ sample), Ling and colleagues [39] found no additional improvements for those randomized to receive buprenorphine with counseling compared to those assigned to buprenorphine alone. In a randomized comparison of methadone only versus methadone combined with counseling in a jail sample, Schwartz and colleagues [70] found no differences between groups in drug-test results collected over a 12-month period following release, an effect that has since been widely replicated without specifying the type of counseling services that are included [46]. This is an important consideration—much like the example of assessments described below, further research is needed to accurately assess the additive value of counseling for MOUD patients, especially for incarcerated populations, and whether requiring it improves outcomes or merely serves as another barrier to treatment access.

### Consideration of co-occurring SUD and mental health disorders

#### What do we know about co-occurring disorders and trauma in correctional settings?

The majority of people who are incarcerated have had lifetime exposures to trauma, including the experience of being incarcerated itself [55], with trauma exposure particularly prevalent in women [44]. In addition, co-occurring disorders, CODs (e.g., mental health and substance use) are common in incarcerated populations. For example, overall half of the people in prison or jails have mental health disorders (MHD, [32]), and 17% of those admitted to jail are estimated to have co-occurring disorders (CODs), with women disproportionately affected [4, 67] Given incarcerated populations’ need for myriad overlapping treatments, understanding how evidence-based practice for treating underlying trauma, MHD, and SUD used in community settings could translate to feasible and effective treatments in carceral settings remains a research gap. However, very few corrections-based SUD treatment programs sufficiently incorporate trauma and/or co-occurring MHD in treatment programming [2, 12].

#### What does trauma-informed care look like in carceral settings?

In particular, while there is increasing understanding of the importance of integrating trauma-informed care (TIC) into carceral treatment and settings [33], we know little of what is effective and feasible in these settings [50]. There is less research emphasis on trauma and CODs (e.g. substance use and mental health disorders) treatment for men in jails/prisons, while there is evidence of the association of trauma exposure and incarceration among men [31, 51]. Intersectional trauma and daily stress exposure for racial/ethnic minority populations add burden to these populations and very little is known about how best to tailor interventions in CJ settings that address the nexus of intersectionality and CODs [41], although there is a prescribed evidence-based principle of providing SUD treatment in CJ settings [53]. While there is some research of COD treatment in criminal justice settings, much of what has been reported has a wide range of outcomes and was largely underpowered [59].

### Lack of agreement over what the goals of SUD treatment in CJ settings should be

It may be common sense that any clinical intervention or policy initiative should have clearly stated goals, but goals of SUD treatment vary across—and even within—criminal justice agencies. In this section, we pose several unanswered questions that merit further debate and study.

#### Is reduced drug use and/or abstinence within the justice setting an appropriate outcome?

In 2002, addiction researcher Thomas McLellan [47] published an influential editorial titled, “Have we evaluated addiction treatment correctly? Implications from a chronic care perspective.” In his article, McLellan pointed out that lower levels of drug use during a treatment episode should be viewed as evidence that the treatment was effective—even if the post-treatment follow-ups showed no improvement. Similar to hypertension patients who show improvements while taking their medication and poorer outcomes after they stop, the fact that symptoms improved during the active phase of treatment suggests that the treatment: (1) had an effect, and (2) should be extended indefinitely for those managing chronic disorders. Additionally, NIDA Director Nora Volkow [84] recently wrote in *Health Affairs Forefront* that “the magnitude of the drug overdose crisis demands out-of-the-box thinking and willingness to jettison old, unhelpful, and unsupported assumptions about what treatment and recovery need to look like. Among them is the traditional view that abstinence is the sole aim and only valid outcome of addiction treatment.” suggesting that we should look at reduced drug use, use of alcohol, or other indicators of changes in use patterns.

#### Is recidivism an appropriate outcome?

Advocates of expanding SUD treatment in CJ settings often argue that doing so also reduces recidivism risk [9]. Randomized trials using an intent-to-treat approach and systematic reviews of more rigorous studies, however, suggest that the effects of CJ-based SUD care on criminal recidivism are minimal and short-lived [15, 60, 71]. Interestingly and perhaps counter-intuitively, problem-solving courts show reductions in recidivism but no changes in drug use patterns [49]. However, if CJ-based SUD treatment can produce meaningful reductions in illicit substance use, is it reasonable to require that it also reduce subsequent criminality? A national, multisite RCT of a drug testing and sanctions approach for probationers found no differences in recidivism [37]. The program was deemed ineffective because of the measured outcome (e.g., recidivism), even though the intervention reduced positive drug tests *by half* relative to probation as usual (see [30]). A recent National Academies of Science report questions the utility of recidivism as the main fixture of justice-related research and argues for other measures of success such as engaged in treatment, housing stability, employment, reduced use, particularly those that support an individual’s pathway to desistance from criminal behavior [69]. This report helps to further interest in varied outcomes that address improved functionality and stability in the community as being valuable and critical.

Relatedly, a recent meta-analysis by Goodley et al. [27], regarding predictors of recidivism following release from custody, found that while there is significant heterogeneity with respect to predictors of recidivism across the extant literature, the authors identified 17 factors with sufficient evidence from the published studies reviewed that were associated with either an increase or decrease in recidivism. Importantly, many of these factors were not necessarily directly related to SUD and were generally categorized by the authors into factors related to: History of Criminal Behavior, Antisocial Personality Pattern, Antisocial Cognitions and Antisocial Companions, all of which have been previously described by Andrews and colleagues [1, 27]. Two additional factors related to increased risk for recidivism identified by Goodley et al. included history of mental illness and race, with the latter posited to reflect the larger context of racial inequality in CJ involvement in the US [27]. It is also important to acknowledge that SUD treatment alone may be unlikely to significantly impact many of the factors identified above by Goodley et al., which is why we believe that recidivism may not be the most appropriate outcome to assess for CJ-involved individuals with an SUD.

### Consideration of mandating SUD treatment

Central to the issue of providing SUD care for those in correctional environments is the role of coercion. In recent decades, many practitioners have justified their support of coerced treatment on the accepted notion that “coerced patients do as well or better than voluntary patients” [48]. As described below, this is an oversimplification that obscures a host of related concerns, including assessment, treatment effectiveness, and the ethics of overriding personal choice.

#### Issues related to voluntary vs. mandatory treatment participation

One of the greatest challenges to the coerced-treatment literature is the synonymous use of terms that are only loosely associated. Our review of the formative literature in this area found that terms such as “coerced,” “compulsory,” “mandated,” “involuntary,” “legal pressure,” and “criminal justice referral” were often used synonymously in the literature, and sometimes the terms are even used interchangeably within the same article [17, 89]. There is some evidence suggesting that mandated SUD treatment may support treatment completion among specific populations, e.g. adults aged 55 and older [63]. In truth, an incarcerated adult with SUD may simultaneously desire SUD treatment and be mandated to participate in it. As a result, many people who are justice-involved and required to receive SUD treatment acknowledge the need for help. In a sample of people on parole receiving psychiatric care, the majority of those who reported no control over admission to the clinic recognized their need for psychiatric treatment [18]. The blurring of these concepts has led to a mixed set of findings regarding the effectiveness of coerced treatment [40]. Another factor (especially from the early literature is that the majority of these studies focused on treatment *retention*, rather than outcomes [88]. Although it may not be surprising that legal pressure to enter and remain in treatment results in lower dropout rates, its value in reducing the severity of substance use behavior remains unclear.

Conspicuously absent from the typical debate over coercion is the *treatment* to which people are coerced to receive. Most SUD treatments offered in prison, and other carceral settings, show no effect unless capitalizing on the selection effects of the subgroup who opt to continue treatment in the community. If we were to assume an effect size of 0.15 of these programs (see [58]), the expected outcomes—with or without coercion—are modest and raise the question whether the coercion or the treatment programs can be justified. Importantly, there is a need to more clearly define what is meant by coercion, particularly within the context of court ordered treatment (e.g. through problem solving courts such as Drug Courts). Coercion can range from legal pressure to engage in treatment to mandates to participate in treatment to placement in a facility, many studies do not differentiate among the various types of coercion but rather indicate that coercion is involvement in treatment without the consent of the patient [89]. In the criminal legal system, this is perplexing because many programs are offered as an alternative to the justice system/incarceration but the individual must still concur to participate in this program. Further work is needed to disentangle two factors that are often conflated: perceived treatment need and level of choice [18].

#### Should we provide SUD treatment to CJ participants who don’t want it?

As mentioned in the previous sections, many of the SUD programming referrals in the CJS are based on the presumed validity of screening and needs assessments and (for those referred to treatment against their wishes) the benefits of coercion. These presumptions are unlikely to be challenged when a facility struggles to find enough participants to fill its designated SUD treatment beds (or when cost of screening/assessment is a prohibitive barrier). Consider, in contrast, a facility with limited SUD treatment slots. Can we justify filling beds with those deemed to have a substance use problem (regardless of their desire to participate) even if it means excluding others who specifically requested it? Evidence would suggest not. Parhar and colleagues [58] reviewed 129 correctional treatment studies and found that mandated treatment was largely ineffective, whereas voluntary treatment showed significant, positive effects. Might we be better off simply offering treatment and other services to those who request it? As suggested above, this remains an unsettled question.

What we do know about the effectiveness of SUD treatment for coerced participants poses an important ethical question: Does the existing evidence for psychosocial treatment justify overriding individual choice in whether to participate? After conducting a systematic review of compulsory treatment evaluations in several countries, Werb and colleagues [86] found that 90% were associated with either null or deleterious effects on drug use and recidivism outcomes. Citing concerns over the documentation of human rights abuses in these settings, the authors concluded: “In light of the lack of evidence suggesting that compulsory drug treatment is effective, policymakers should seek to implement evidence-based, voluntary treatment modalities in order to reduce the harms of drug use” (p. 8). None of these individual studies—or even reviews—can be considered dispositive, but at the very least it is clear that existing evidence provides an insufficient basis for the use of coerced SUD treatment. As SUD treatment itself becomes more effective, it will be interesting to re-examine the potential role of coercion. An alternative method for overcoming patient resistance is to offer incentives for desired drug-use outcomes. This approach—known as “contingency management” (CM)—involves providing small, incremental payments for negative drug-test results and no requirements to attend counseling sessions. Although the CJS has been slow to adopt CM, a recent analysis by the Washington State Institute for Public Policy [85] revealed that CM produced the highest cost–benefit ratio of all of the dozens of interventions reviewed.

### Limitations of political and policymaking leadership: implications for SUD treatment

While this paper speaks to additional research and knowledge gaps relative to SUD treatment in correctional systems, another shortcoming of the current legal-health systems rests in the lack of integration of what *is* evidence-based into formal policies and practices implemented through executive/legislative policy or action. Policymaking and policy implementation often are slow and incremental, with significant gaps between initial formulation and actual implementation. Advancement of MOUDs in correctional settings is one example, though the gap between what national leaders in the field recommend and policies actually put into place is significant, though improving somewhat in the past 1–2 years. For example, a recent study surveyed state prisons in states with disproportionate opioid overdose mortality and found that while all prison systems within the states surveyed reported offering at least one medication to treat OUD, only 7% of the 538 individual prisons within the states offered all three medication types (methadone, buprenorphine and naltrexone); 61% of the 538 prisons did not offer any type of MOUD [72]. A recent study on medications used in a nationally representative same of 832 problem solving courts reported that while 86% of the courts would authorize the use of medications, only 14% of those with an opioid use disorder were on MOUD [20]. Other models employing evidence-based cognitive-behavioral treatment (CBT are in place, including in the Federal Bureau of Prisons’ non-residential treatment program (a 12-week course for those serving short sentences and/or who are transitioning back to the community as part of their SUD treatment, Federal Bureau of Prisons, n.d.). Georgia’s Department of Corrections also offers a CBT model for some individuals with SUD, incorporating moral reconation therapy, Motivation for Change, and other approaches (Georgia Department of Corrections, n.d.).

#### Why are uptake and acceptance of current best practices for SUD treatment in correctional facilities lagging by legislative and executive leaders?

Organizations as diverse as the National Institute on Drug Abuse, American Society of Addiction Medicine, American Medical Association, National Governors Association, and National Commission on Correctional Health Care (NCCHC) have endorsed MOUD treatment as a best practice. The Legislative Analysis and Public Policy Association even developed model legislation for states and localities to use as a foundation for introduction of legislation to provide MOUD in correctional settings [38] through a panel co-chaired by Regina Labelle, formerly acting Director of the White House Office of National Drug Control Policy (ONDCP). Yet, until very recently, MOUD treatment in prisons and jails has been relatively rare, and few such programs have included coverage of two or more approved MOUDs consistent with established best practices [52]. The lack of widespread coverage of MOUDs and inconsistent administration of them, despite the relatively high proportion of incarcerated individuals with OUD/SUD, occur for several reasons. This is the case, even though the U.S. Department of Justice and recent court rulings have established that a correctional facility’s failure to provide such care may violate the “cruel and unusual punishment” language in the Eighth Amendment to the U.S. Constitution [61, 76, 82], not to mention whether such care is administered in accordance with established standards and best practices. For one, the standards established for correctional health care by the NCCHC, while advancing best practices, are entirely voluntary [68], so conditions, standards, and metrics surrounding healthcare for incarcerated individuals at the state and local level are inconsistent and scattered.

Some programs that do offer MOUD suffer operationally due to lack of trained providers, misunderstanding or lack of information within correctional institutions, and other factors [66, 77]. Even fewer provide counseling or complementary mental health services, and very few programs provide any kind of significant treatment for other SUD. Funding is another concern. While the average annual state budget for an incarcerated individual was $5720 in 2015, the figure was wildly divergent across states, from nearly $20,000 in California to about $2200 in Louisiana [62]. This discrepancy translates to significant variations in staff and expertise dedicated to health care, notably mental health/SUD treatment, for incarcerated individuals, quality of care, and health status of individuals once they are released from correctional facilities [8]. In addition, while incarcerated individuals and pretrial detainees are the only Americans with Constitutionally guaranteed rights to “adequate medical care” [14], what constitutes adequate care is less clear, including in terms of funding. Incarcerated individuals generally are limited as to sources of funding, Medicaid in particular, that may be provided for their medical care under the 1965 Medicaid Inmate Exclusion Policy [13]. Six states have applied to the federal government for waivers that would allow them to cover some Medicaid-eligible services outside of the exclusion policy for individuals nearing release; these applications are under review by the Center for Medicare and Medicaid Services [28].

Another step toward expanding best practice-based OUD treatment in correctional facilities is being pursued on the legal front. The U.S. Department of Justice has challenged existing MOUD policy in correctional facilities, citing potential violations of the Americans with Disabilities Act (ADA) [80]. Because the ADA protects individuals with disabilities, and individuals in treatment/recovery for OUD who are not engaged in illegal drug use are deemed to have a disability [81], denial or non-provision of MOUD treatment for those with SUD/OUD diagnosis, the Department argues, violates the law. The Department has issued guidance on protections for individuals with OUD under the ADA and taken action in several cases through its Civil Rights Division against healthcare institutions, professional boards, state courts, and private employers that have denied treatment and services to justice-involved individuals [83].

#### Progress around MOUD is promising for future treatment options

While the pace of adoption has long been lagging, coordinated efforts advancing evidence-based practices, at least around MOUD treatment in correctional facilities, are producing results. Since the beginning of 2020, legislation in at least a dozen states has established or expanded access to MOUD, and most states now have at least one correctional facility that provides some form of MOUD [87]. Additionally, at least 28 states have, through executive action or agency rulemaking, advanced SUD treatment for incarcerated individuals, mostly in state prisons [57]. Under Labelle’s leadership as Acting Director of the ONCDP, the first Statement of Drug Policy Priorities of the Administration affirmed as its top priority expanding access to evidence-based treatment, including for incarcerated individuals [56].

A few states, Massachusetts and Maryland notably, have recently passed legislation that incorporates evidence-based best practices for OUD treatment (e.g., inclusion of at least one medication from all three MOUD classes; and provision of mental health/counseling services as part of treatment [24, 45]. Others, like Rhode Island, have incorporated evidence-based research across the state corrections system [77] in prisons and in the community. This model has been adopted by a correctional facility in neighboring Massachusetts [11], though most state programs still lag far behind these models.

#### The need for state and federal support of replication studies

In addition, we strongly support the importance of reproducibility within CJ research. It is unfortunate that often only a single study is conducted in single (or relatively few), correctional facilities. A benchmark of science is the extent to which research findings are reproducible and transportable. Most of us are familiar with publication bias, the phenomenon that studies with null findings are less likely to be published in the scientific literature [23]. However, we note that funding bias, a variant on what Knottnerus and Tugwell term the “research-agenda bias”, is also an important contributor to the lack of reproducibility in CJ data. For example, many grant applications emphasize the importance of innovation. Yet, often studies that seek to replicate the findings of prior research to develop a more robust evidence base are not considered innovative (and often not funded). This means that as researchers, we are continually placed in a position where we cannot make firm conclusions or recommendations based on available evidence because the studies designed to produce that evidence are not prioritized for funding. The importance of replicating the foundational studies in this field cannot be overstated. Camerer and colleagues [7] replicated 21 social science studies published in the journals *Nature* and *Science* between 2010–2015 using much larger sample sizes and found that only 62% produced a significant effect in the same direction as the original study. They also found that the effect sizes were approximately one half of those reported in the original studies. It is quite plausible that replicability of studies published in lower-tier journals would be even lower [7].

It will be important to take the lessons learned around adoption and implementation of MOUD in correctional settings and apply these to other treatment modalities. However, policy makers have another important role in this space, namely to promote SUD related research within correctional institutions. Often correctional facilities put into place significant barriers to conducting research in their facilities, including banning researchers or withholding important data around current policies, practices and procedures [36]. Policymakers can play an instrumental role in promoting more research and transparency within correctional institutions in order to generate the necessary evidence based for effective SUD treatment in correctional settings.

### A proposed list of research priorities

We conclude with a set of study topics that, if rigorously conducted, would substantially advance our understanding of how best to deliver SUD treatment to patients in prison and jail settings. We are aware that some of these topics have been studied before, but they are revisited here because the existing body of literature still lacks the necessary rigor, consistency in findings, and sufficient studies to provide a solid empirical foundation to guide clinical practice or policy decisions in this critical public health domain.

As mentioned above, more research is needed regarding SUD treatment dose response. This should be assessed in correctional settings using community-based standards for appropriate treatment duration through randomized or prospective studies rather than correlational observation studies. For treatment modalities that include pharmacotherapy, such as MOUD, limited evidence exists with respect to ancillary psycho-education or counseling components that can accompany pharmacotherapy to optimize treatment effectiveness. Furthermore, we have highlighted in this article that most individuals involved in the criminal justice system have complex co-occurring disorders, so more research is needed in order to understand which community-based evidence-based practices for COD are feasible and effective within carceral settings. Relatedly, research on how treatment modalities can be adapted or modified to integrate a more trauma-informed approach are needed in order to address the significant burden of trauma that many CJ-involved individuals endorse. Finally, given that 60% of incarcerated adults are people of color [29] and this is a reflection of a system that disproportionately surveils, arrests, and sentences racial and ethnic minorities despite similar drug use prevalence [22], more data are needed with respect to culturally tailored SUD treatment modalities that will be acceptable among diverse racial and ethnic correctional populations.

We also strongly recommend a rethinking of currently accepted goals for treatment outcomes for correctional populations. For example, the primary outcome many correctional institutions prioritize for criminal justice involved individuals with SUD is recidivism. To date, findings on the impact of SUD treatment on recidivism are equivocal. This begs the question as to whether or not recidivism is the most appropriate SUD treatment outcome among CJ-involved populations. As the National Academy of Sciences recent report on recidivism and success measures, other measures of functionality and well-being are worthy outcomes [69]. Future studies need to better evaluate the extent to which involvement in SUD treatment, both during incarceration and after release, directly impacts risk for re-arrest, independent of other extenuating factors which place individuals at greater risk for continued interaction with law enforcement (e.g., failure to pay fees and fines). It is also worth asking the question: is it reasonable to require that CJ-based treatment produce reduced drug use (or abstinence) beyond the period of incarceration and/or community supervision (e.g. parole or probation)? The field of SUD recovery research, while growing, is still nascent, and more data are needed to better understand the non-CJ modalities that can best facilitate sustained recovery within CJ settings.

There has been considerable debate on whether or not to mandate SUD treatment for CJ populations. However, “coerced” status is often inferred from one’s CJ status rather than directly measured through individual interviews. More research is needed that: (1) more accurately measures perceived coercion among individuals with SUD in CJ settings; and (2) whether the outcomes of SUD treatment justify overriding personal choice and, in some cases, displacing individuals with SUD who seek treatment voluntarily. Increasingly, CJ-based services, including SUD treatment, are informed by algorithms that incorporate specific individual attributes, including demographics, criminal and substance use history, to predict needs and likely treatment outcomes [35]. However, this can negate individual choice and remove autonomy among individuals with respect to what they perceive they actually need, especially with respect to ancillary services such as employment and housing, in order to achieve and sustain long-term recovery.

## Conclusion

In conclusion, our discussions, literature overview, and consultation with experts in the field have identified clear and important gaps in the current evidence base with respect to treatment effectiveness within CJ settings. While there are important clinical considerations during treatment delivery within prisons and jails (e.g., space restrictions, security, confidentiality, etc.), it is difficult to make firm clinical recommendations without sufficient effectiveness research. One fundamental aspect that is lacking from the extant literature is a natural history of treatment related outcomes during incarceration. Such a longitudinal investigation could enroll participants prior to incarceration and conduct follow-up both during and upon release from incarceration, to provide rich contextual data about treatment delivery and effectiveness across the CJ continuum. This kind of research would provide important insight into individual characteristics (e.g., motivation, treatment modality preferences, treatment completion rates, etc.) as well as institutional and environmental factors (e.g., appropriate staffing, space limitations for individual treatment sessions, distribution of medications, etc.).

As we stated at the beginning of this paper, the issues raised (and accompanying research priorities proposed) are undoubtedly incomplete. But is it our hope that others will join the healthy process of questioning the existing state of the CJ-based SUD treatment research, what should be re-examined, and how we can lay a stronger foundation for the future.

## Data Availability

Not applicable.

## Abbreviations

CJ: Criminal justice
SUD: Substance use disorder
